# *OsNAC129* Regulates Seed Development and Plant Growth and Participates in the Brassinosteroid Signaling Pathway

**DOI:** 10.3389/fpls.2022.905148

**Published:** 2022-05-16

**Authors:** Su-Kui Jin, Ming-Qiu Zhang, Yu-Jia Leng, Li-Na Xu, Shu-Wen Jia, Shui-Lian Wang, Tao Song, Ruo-An Wang, Qing-Qing Yang, Tao Tao, Xiu-Ling Cai, Ji-Ping Gao

**Affiliations:** ^1^Jiangsu Key Laboratory of Crop Genomics and Molecular Breeding/Key Laboratory of Plant Functional Genomics of the Ministry of Education/Jiangsu Key Laboratory of Crop Genetics and Physiology/Jiangsu Co-Innovation Center for Modern Production Technology of Grain Crops, College of Agriculture, Yangzhou University, Yangzhou, China; ^2^National Key Laboratory of Plant Molecular Genetics, CAS Center for Excellence in Molecular Plant Sciences, Shanghai Institute of Plant Physiology and Ecology, Chinese Academy of Sciences, Shanghai, China; ^3^University of Chinese Academy of Sciences, Beijing, China; ^4^Innovation Academy for Seed Design, Chinese Academy of Sciences, Beijing, China

**Keywords:** grain size, starch biosynthesis, NAC transcription factor, brassinosteroids, abscisic acid

## Abstract

Grain size and the endosperm starch content determine grain yield and quality in rice. Although these yield components have been intensively studied, their regulatory mechanisms are still largely unknown. In this study, we show that loss-of-function of *OsNAC129*, a member of the NAC transcription factor gene family that has its highest expression in the immature seed, greatly increased grain length, grain weight, apparent amylose content (AAC), and plant height. Overexpression of *OsNAC129* had the opposite effect, significantly decreasing grain width, grain weight, AAC, and plant height. Cytological observation of the outer epidermal cells of the lemma using a scanning electron microscope (SEM) revealed that increased grain length in the *osnac129* mutant was due to increased cell length compared with wild-type (WT) plants. The expression of *OsPGL1* and *OsPGL2*, two positive grain-size regulators that control cell elongation, was consistently upregulated in *osnac129* mutant plants but downregulated in *OsNAC129* overexpression plants. Furthermore, we also found that several starch synthase-encoding genes, including *OsGBSSI*, were upregulated in the *osnac129* mutant and downregulated in the overexpression plants compared with WT plants, implying a negative regulatory role for *OsNAC129* both in grain size and starch biosynthesis. Additionally, we found that the expression of *OsNAC129* was induced exclusively by abscisic acid (ABA) in seedlings, but *OsNAC129*-overexpressing plants displayed reduced sensitivity to exogenous brassinolide (BR). Therefore, the results of our study demonstrate that *OsNAC129* negatively regulates seed development and plant growth, and further suggest that *OsNAC129* participates in the BR signaling pathway.

## Introduction

Rice is one of the most important cereal crops, providing a staple food for more than half of the world’s population. Thus, grain yield and grain quality are the most two critical issues in rice production. Grain yield is generally determined by tiller number (also referred as panicle number per plant), grain number per panicle, and grain size. Grain size, in turn, is determined by a combination of grain length, grain width, and grain thickness. Grain size is a complex quantitative trait controlled by multiple genes. To date, at least 280 grain size-related genes (TO:0000397) have been reported and accessed in the China Rice Data Center.^1^ These cloned genes have provided considerable insight into the individual molecular basis of grain size and shape regulation. Based on the results of previous studies, grain size is regulated by multiple pathways including the G-protein signaling pathway, the ubiquitin-proteasome pathway, the IKU pathway, the MAPK signaling pathway, the phytohormone signaling pathway, and the transcriptional regulatory pathway (Zuo and Li, 2014; Li and Li, 2016; Li et al., 2019). In any case, these pathways ultimately control seed size by influencing cell proliferation or/and cell expansion in maternal tissues or endosperm growth.

Among these pathways, the brassinosteroid (BR) pathway and transcriptional regulatory pathway are the most widely studied and well-characterized pathways. BRs are a class of polyhydroxysteroid phytohormones that are essential for the proper regulation of multiple physiological processes during plant growth and development (Brosa, 1999; Müssig, 2005; Gudesblat and Russinova, 2011; Ackerman-Lavert and Savaldi-Goldstein, 2020). Many studies have shown that BRs also participate in grain size regulation. For example, the BR-deficient mutants *dwarf11* (*d11*) and *dwarf2* (*d2*) and the BR-insensitive mutants *dwarf61* (*d61*/*bri1*) and *08sg2* (*bak1*) all produce small and short grains, whereas overexpression of *OsBZR1* increases grain length, grain width, and grain weight (Hong et al., 2005; Tanabe et al., 2005; Morinaka et al., 2006; Zhu et al., 2015; Wu et al., 2016; Yuan et al., 2017). In addition to these direct BR components, other indirect components such as *GL3.1*, *SLG*, *SG1*, *GSE5*, *BU1*, *GS5*, and *DLT* all participate in the regulation of grain size through the BR signaling pathway (Tanaka et al., 2009; Li et al., 2011; Hu et al., 2012; Nakagawa et al., 2012; Qi et al., 2012; Tong et al., 2012; Zhang et al., 2012; Feng et al., 2016; Duan et al., 2017; Liu et al., 2017; Gao et al., 2019). Transcriptional regulation of grain size is another important pathway because many of the identified grain-size-related genes encode transcription factors. For example, it has been reported that *GLW7* (*OsSPL13*) and *GW8* (*OsSPL16*), genes that encode the plant-specific transcription factor (TF) SQUAMOSA PROMOTER BINDING PROTEIN-LIKE, positively regulate grain length and yield by promoting cell expansion and cell division in the grain hull, respectively (Wang et al., 2012a; Si et al., 2016). Elevated expression of *GL7* (*GW7/SLG7*), a gene that encodes a TON1 RECRUITING MOTIF (TRM)-containing protein, leads to slender grains, but further studies are needed to clarify whether *GL7* (*GW7/SLG7*) controls grain size through cell proliferation or cell expansion (Wang et al., 2015a,b; Zhou et al., 2015). *GS2* encodes a plant-specific transcription factor called GROWTH-REGULATING FACTOR 4 (OsGRF4), which regulates grain size mainly by increasing cell expansion and slightly promoting cell proliferation in the spikelet hull (Che et al., 2015; Duan et al., 2015; Hu et al., 2015; Li et al., 2016; Sun et al., 2016).

Additionally, these pathways do not always act in isolation, but interact with one another to shape and fine-tune grain size. For example, two atypical bHLH domain transcription factors without DNA-binding activity are encoded by *OsPGL1* and *OsPGL2*/*OsBUL1* and function as inhibitors of a typical DNA-binding bHLH TF OsAPG through heterodimerization (Heang and Sassa, 2012a,b; Jang et al., 2017; Jang and Li, 2017). Overexpression of both of these genes leads to increased grain length caused by increased cell length. Further study revealed that *OsPGL1* and *OsPGL2*/*OsBUL1* are BR-related genes, even though *OsPGL1* is not BR-inducible (Heang and Sassa, 2012a,b; Jang et al., 2017; Jang and Li, 2017). Thus, *OsPGL1* and *OsPGL2*/*OsBUL1* function in the transcriptional regulation and BR pathways. Recently, it was reported that the MAPK signaling pathway component OsMKK4 and its substrate OsMAPK6 in the OsMKKK10-OsMKK4-OsMPK6 cascade positively regulate grain size by promoting cell division of the spikelet hulls (Guo et al., 2018; Xu et al., 2018). Moreover, both of these proteins affect BR responses and the expression of BR-related genes, indicating that there is crosstalk between the BR and MAPK pathways.

Storage starch, a mixture of two glucose polymers called amylose and amylopectin, accounts for 90% of the endosperm dry weight (Keeling and Myers, 2010; Zeeman et al., 2010), and thus has a very large effect on grain yield and quality. The starch biosynthesis pathway in the endosperm of cereal species is highly conserved. After decades of investigation, the key enzymes including ADP-glucose pyrophosphorylase (AGPase), granule bound starch synthases (GBSSs), soluble starch synthases (SSs), starch branching enzymes (SBEs), debranching enzymes (DBEs), and starch phosphorylases (PHOs) involved in this pathway have been fully characterized and are well reviewed in the literature (Jeon et al., 2010; Zhu et al., 2020; Huang et al., 2021). Among these enzymes, AGPase is an allosterically rate-limited enzyme responsible for synthesis of the starch substrate ADP-glucose (ADPG); GBSSs, especially GBSSI, are the only enzymes responsible for amylose synthesis; SSs, SBEs, and DBEs are cooperatively responsible for the synthesis of amylopectin; PHOs are thought to function in starch initiator synthesis (Jeon et al., 2010; Zhu et al., 2020; Huang et al., 2021). When these synthases involved in starch synthesis are dysfunctional, seed development and the filling process are disturbed, leading to abnormal seed phenotypes. In addition, the direct or indirect transcriptional regulators of starch synthase encoding genes also have significant influence on grain starch content and seed development. To date, however, only a few starch biosynthesis regulators have been functionally identified. For example, bZIP TF members including OsbZIP58, OsbZIP33, OsbZIP76, O2, ZmbZIP22, and ZmbZIP91 are all reported to directly bind and activate/suppress one or more starch synthase-coding genes (Yang et al., 2001; Wang et al., 2013; Chen et al., 2016; Zhang et al., 2016; Dong et al., 2019; Niu et al., 2020). In addition to the bZIP TFs, recently, a number of NAC TFs such as OsNAC20, OsNAC26, OsNAC127, OsNAC129, ZmNAC36, ZmNAC128, ZmNAC130, and TaNAC019 have been shown to regulate starch biosynthesis and/or storage protein synthesis (Zhang et al., 2014, 2019b; Peng et al., 2019; Wang et al., 2020; Gao et al., 2021; Ren et al., 2021).

NAM, ATAF1/2, and CUC2 (NAC) TFs comprise one of the largest plant-specific TF protein families and are charactered by the presence of a conserved NAC domain at the N-terminus and a highly variable transcriptional regulation region in the C-terminus (Olsen et al., 2005; Nuruzzaman et al., 2010, 2013). The NAC domain is further separated into five sub-domains (A–E), each of which has a specific biological function: sub-domain A plays a role in dimer formation with other TFs; sub-domains B and E are relatively divergent and may be related to NAC protein functional diversity; sub-domains C and D are responsible for binding to DNA (Ernst et al., 2004; Olsen et al., 2005; Jensen et al., 2010; Chen et al., 2011; Puranik et al., 2012). NAC TFs play multiple vital roles in plant growth and development, the stress response, plant hormone signal transduction, and leaf senescence (Singh et al., 2021). It has been reported that there are at least 151 NAC TF genes in the rice genome, and that nine of them are seed-specific, indicating a wider range of functions for NAC TFs in seed development (Fang et al., 2008; Nie et al., 2013).

In this study, we characterized a seed-specific NAC TF gene *OsNAC129* (*LOC_Os11g31380*). Loss-of-function of *OsNAC129* led to increased grain length, grain weight, apparent amylose content (AAC), and plant height, while transgenic plants overexpressing *OsNAC129* displayed essentially the opposite phenotypes. Further study revealed that *OsNAC129* negatively regulates grain size and AAC by inhibiting the expression of genes related to grain size and starch synthesis. Moreover, we showed that *OsNAC129* participates in the BR signaling pathways.

## Materials and Methods

### Plant Materials and Growth Conditions

For overexpression of *OsNAC129*, the full length DNA fragment including 2 kb of promoter sequence upstream of the start codon, the open reading frame (ORF) from the start codon to the stop codon, and 1 kb downstream of the stop codon was amplified from genomic DNA of the *japonica* rice (*Oryza sativa* L.) cultivar “Nipponbare” by PCR. The DNA fragment was then introduced into pCAMBIA 1300 by recombinase. For the promoter-driven GUS reporter, 2 kb of DNA sequence upstream of the start codon was amplified from genomic DNA of “Nipponbare” by PCR, and the DNA fragment was introduced into the pCAMBIA 1300-GN vector by recombinase. All of the recombinant plasmids were introduced into *Agrobacterium tumefaciens* strain EHA105 and used to infect callus of “Nipponbare” to obtain transgenic plants. The *japonica* rice cultivar “Dongjin” and the *osnac129* T-DNA mutant (PFG_3A-60140) were obtained from Pohang University of Science and Technology, Korea (Jeong et al., 2002). Paddy field conditions: plants were grown at the experimental stations in Shanghai (121°24′ E, 31°00′ N) during the summer season, or at Lingshui (110°00′ E, 18°31′ N) during the winter season under natural conditions mainly for phenotypic analysis, detection of gene expression, and seed production. Plants were grown in the greenhouse at 28°C with an 11-h day/13-h night photoperiod mainly for seedling cultivation and for gene expression experiments.

### DNA and RNA Isolation and qRT-PCR Analysis

Plant DNA and plasmid DNA isolation was performed using a plant DNA isolation kit (Tiangen Biotech) and a plasmid DNA extraction kit (Tiangen Biotech), respectively, following the manufacturer’s instructions. For RNA isolation, all tissues were collected fresh, flash-frozen in liquid nitrogen, and total RNA was extracted using a plant total RNA isolation kit (Tiangen Biotech) following the manufacturer’s instructions. First-strand cDNA was synthesized using a reverse transcription kit (TAKARA) as directed by the manufacturer. For qPCR, 2 μl of cDNA was used as template in 20 μl reaction volumes, and the qPCR amplifications were performed using TB Green® Premix Ex Taq™ II (Tli RNaseH Plus; TAKARA) on a LightCycler® 480 instrument (Bio-Rad). The rice *UBIQUITIN10* (*OsUBQ10*) or *ACTIN1* genes were used as the internal controls for normalization of gene expression. All oligonucleotide primers used in this study are given in Supplementary Table 1.

### Southern Blot Analysis

Southern blot analysis of the *osnac129* mutant was performed as described previously (Liu et al., 2020).

### GUS Assays

Histochemical analysis of GUS activity was performed as described in a previous study with slight modification (Edwards et al., 1990). Briefly, the tissues of transgenic plants expressing the *OsNAC129* pro::GUS construct were collected and fixed in 90% acetone aqueous solution for 15 min on ice. The fixative solution was discarded and the tissues were washed twice with deionized water. Subsequently, the tissues were immersed in the GUS staining solution and allowed to stain overnight at 37°C. The GUS staining solution was discarded the following day and ethanol was added to decolorize the tissues so that the GUS staining signal could be observed and imaged.

### Determination of Endosperm Starch Content

Total starch content (TSC) and apparent amylose content determination were performed as described previously (Wang et al., 2013).

### Seed Phenotype Observation and Lemma Outer Epidermis Cell Measurement

Grain size of mature seeds harvested from transgenic plants was measured using the WSeen SC-G automatic seed testing system and thousand-grain weight analysis system (Hangzhou WSeen Detection Technology Co., Ltd., China) following the manufacturer’s operating instructions. There were three biological replicates per sample and each replicate contained more than 300 seeds. Lemma outer epidermis cell measurement was performed as previously described (Heang and Sassa, 2012a). Starch grain morphology observation was performed as described previously (Fu and Xue, 2010).

### Plant Hormone Induction Assay

About 1-week-old “Nipponbare” seedlings were submerged in distilled water containing the various plant hormones, and distilled water without added plant hormones was used as the control. The hormone-treated samples were collected at different times and immediately frozen in liquid nitrogen. All the collected samples were then used in qRT-PCR assays to determine the relative levels of *OsNAC129* expression. RNA isolation, reverse transcription, and qRT-PCR amplifications were performed as described above.

### BL Sensitivity Assay

About 2-week-old “Nipponbare” and *OsNAC129*-OE seedlings were treated with brassinolide (BL) at three concentrations (1, 5, and 50 μM) for 24 h. Sterile water treatment was used as the control. The seedlings were then imaged and the leaf angles calculated by ImageJ following the manufacturer’s instructions.

## Results

### *OsNAC129* Is Highly Expressed in Immature Rice Seeds

**Figure 1:**
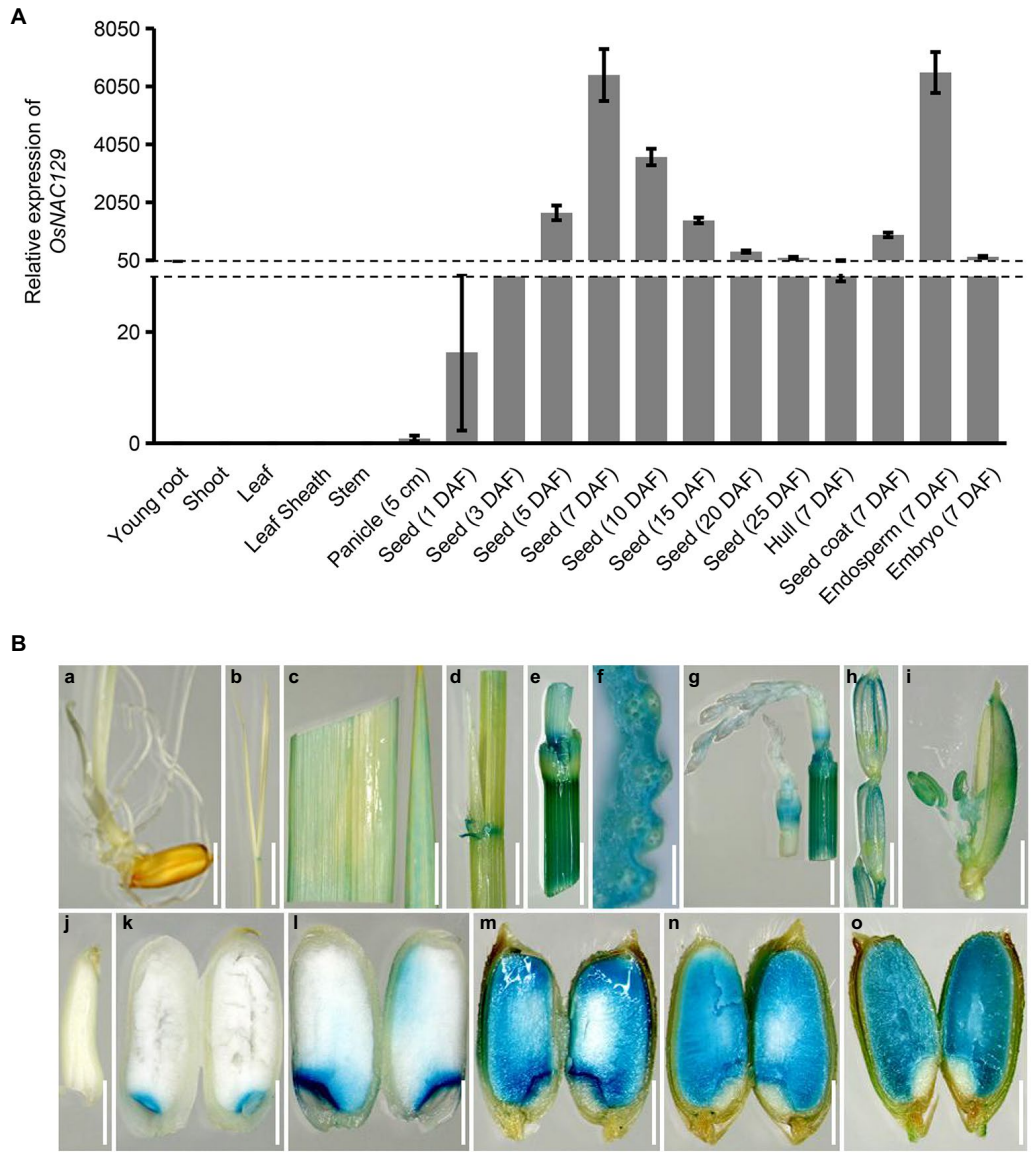
*OsNAC129* is highly expressed in immature seeds. **(A)** Expression profiles of *OsNAC129* in “Nipponbare.” Total RNA was extracted from roots and shoots at 7 days after germination, from leaves, leaf sheathes, stems, and young panicles before flowering, and from developing seeds from 1 to 25 days after fertilization (DAF). *Actin1* was used as the internal control for normalization of gene expression. Data are means ± SD of four biological replicates. **(B)** *OsNAC129* promoter-GUS expression patterns in transgenic plants. GUS expression in root **(a)** and shoot **(b)** at 7 days after germination, flag leaf **(c)**, leaf sheath **(d)**, stem **(e)**, and cross section of stem **(f)** at heading date, younger panicle **(g)**, older panicle **(h)**, flowering spikelet **(i)**, and endosperm at 3 DAF **(j)**, 5 DAF **(k)**, 7 DAF **(l)**, 10 DAF **(m)**, 15 DAF **(n)**, and 25 DAF **(o)**. Scale bars **(a–e, g, and h)** = 5 mm; scale bar **(f)** = 1 mm; scale bars **(i–o)** = 2.5 mm.

It was previously reported that nine NAC TFs gene show seed-specific expression patterns, and *OsNAC129* was one of them (Fang et al., 2008; Nie et al., 2013). In order to comprehensively reveal the functions of *OsNAC129*, we determined its expression profile by qRT-PCR in various tissues and developmental stages in the *japonica* rice cultivar “Nipponbare” (NIP). The results showed that *OsNAC129*-specific mRNA was almost undetectable in vegetative tissues including roots and shoots at 7 days after germination, and in the flag leaves, leaf sheaths, and stems at heading (Figure 1A). Additionally, the expression level was also very low in panicles early in development (Figure 1A). Expression of *OsNAC129* increased rapidly in immature seeds after fertilization and peaked at 7 days after fertilization (DAF), then gradually decreased with development (Figure 1A), which is consistent with the grain filling process. In addition, we confirmed that the highest expression in seeds was mainly detected in the endosperm (Figure 1A). We also found that *OsNAC129* was highly expressed in the spikelet hull, seed coat, and embryo (Figure 1A), implying its potential functions in the regulation of seed development.

To better characterize the expression of *OsNAC129*, we also generated transgenic plants by transforming an *OsNAC129* pro::GUS construct into the cultivar NIP. A histochemical GUS activity assay was then performed on tissues from the transgenic plants. The results showed that GUS staining signals could not be detected in roots and shoots at 7 days after germination except in the lamina joints (Figures 1Ba,b). Weak GUS staining was detected in leaves and leaf sheaths and relatively stronger GUS staining was detected in lamina joints before flowering (Figures 1Bc,d). Additionally, we detected strong GUS staining in the stem nodes and internodes (Figures 1Be,f). Moreover, as the panicles developed to the flowering stage, the GUS staining signals became stronger, and the stamen was also stained (Figures 1Bg–i). After flowering and fertilization, the embryo and endosperm begin to form. GUS staining was also undetectable until 5 DAF during the early stage of seed development (Figures 1Bj,k), and the staining was mainly concentrated at the junction of the embryo and endosperm (Figures 1Bk,l). Also, GUS staining became progressively stronger and was distributed throughout the entire endosperm after 7 DAF (Figures 1Bk–o). In conclusion, these results confirmed that *OsNAC129* is a universally expressed gene with the highest level of expression detected in immature seeds, suggesting a key role in seed development and plant growth regulation.

### Loss-of-Function of *OsNAC129* Leads to Increases in Grain Size, Apparent Amylose Content, and Plant Height

**Figure 2:**
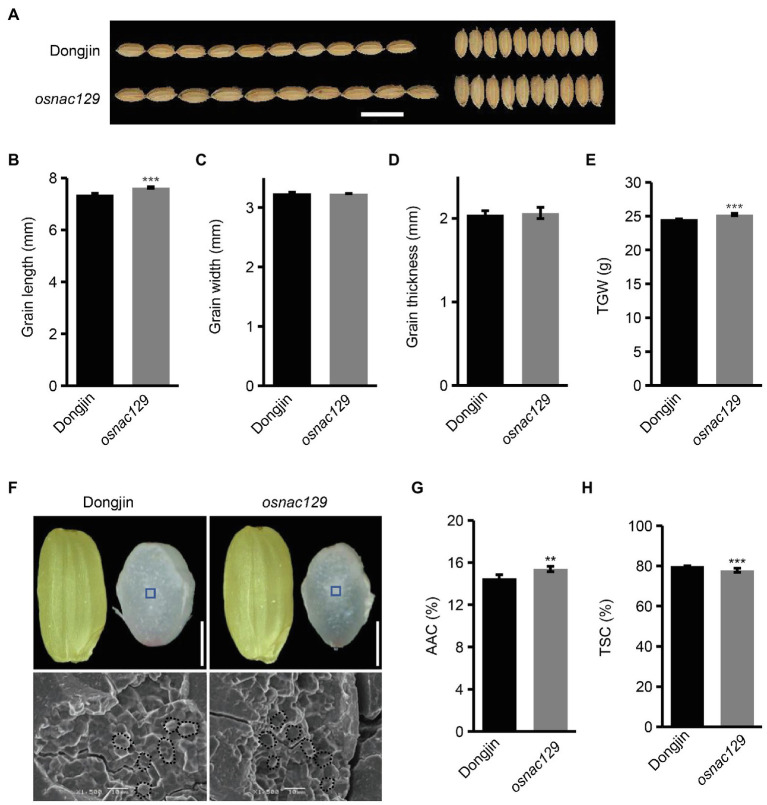
The *osnac129* mutant has increased grain size and apparent amylose content. **(A)** Phenotypic observation of *osnac129* grain at maturity. Scale bar = 10 mm. **(B–E)** Show the grain length, grain width, grain thickness, and 1,000-grain weight (TGW) of mature seeds, respectively. Data are means ± SD of three replicates. **(F)** Brown rice grains and cross sections of grains (upper panel, scale bars = 2.5 mm), and electron micrographs of starch granules (lower panel, scale bars = 10 μm) from the wild-type (WT) and *osnac129* mutant. Blue boxes in the upper panels indicate the central area of the mature endosperm, where starch granules were analyzed by scanning electron microscope (SEM), and areas in the bottom panels delineated by dotted lines indicate starch grains. **(G,H)** Show the apparent amylose content (AAC) and total starch content (TSC) of grains from the WT and *osnac129* mutant, respectively. Data are means ± SD of five replicates. ^**^p < 0.01, ^***^p < 0.001 as determined by Student’s *t*-test.

Subsequently, we obtained a T-DNA insertion mutant called *osnac129* (PFG_3A-60140) from the Rice T-DNA insertion sequence database (RISD) collection. According to description of this mutant, a T-DNA is inserted into the third exon of *OsNAC129* (Supplementary Figure 1A). The T-DNA insertion site in the *osnac129* mutant was verified by a PCR assay using gene- and T-DNA-specific primers (Supplementary Figures 1A,B). Homozygous mutant plants were further isolated from segregating progeny (Supplementary Figure 1B). Moreover, a Southern blot assay confirmed that there was only a single T-DNA insertion in this mutant (Supplementary Figure 1C), indicating a specific mutation in *OsNAC129*. The relative transcription of *OsNAC129* was also found to be significantly reduced (Supplementary Figure 1D). Hence, *osnac129* is a loss-of-function mutant. We next observed the phenotypes of *osnac129* mature seeds. The results of this analysis showed that grain length and grain weight of *osnac129* mutant seeds were increased compared with WT plants (Figures 2A,B,E), while grain width and grain thickness showed no obvious change (Figures 2A,C,D). These results further confirmed that *OsNAC129* negatively regulates grain size, especially grain length. Likewise, the appearance of the endosperm in *osnac129* mutant seeds was semi-transparent and non-chalky, indistinguishable from WT except for the slender seed shape (Figure 2F). Scanning electron microscope (SEM) images revealed that the starch granules observed in cross sections of *osnac129* endosperm were closely packed and polyhedral, similar to WT (Figure 2F). We then measured the starch content of *osnac129* and WT seeds, and found that the AAC was increased but that the TSC was reduced in *osnac129* endosperm compared with WT (Figures 2G,H). Collectively, these results showed that *OsNAC129* participates in the regulation of both grain shape and starch biosynthesis in rice.

Considering that *OsNAC129* expression was also detected in stems, we sought to determine whether *OsNAC129* plays any roles in vegetative growth and development. As a result, we found that the shoots of *osnac129* mutant plants were longer than in WT plants at the seedling stage, while root lengths were reduced compared to WT (Supplementary Figures 2A–C). Moreover, plant height in the *osnac129* mutant was also increased at maturity compared with WT (Supplementary Figures 2D,E), but tiller number was not affected. Therefore, these results indicate that *OsNAC129* plays a negative role in regulating plant height in rice.

### Overexpression of *OsNAC129* Leads to Reductions in Grain Size, Apparent Amylose Content, and Plant Height

**Figure 3:**
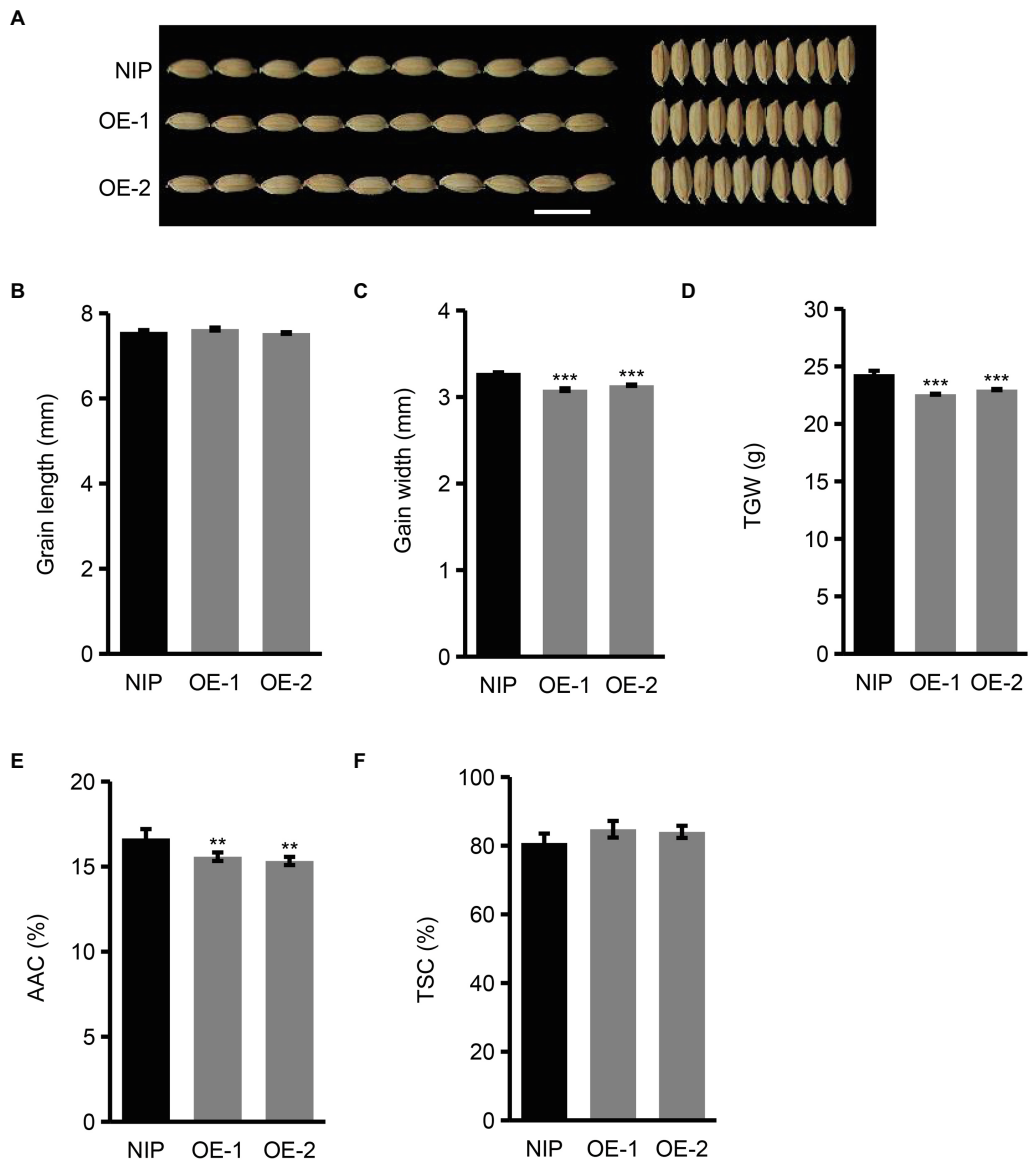
Overexpression of *OsNAC129* leads to reductions in grain size and apparent amylose content. **(A)** Phenotypic observation of WT and OE plants mature seeds. Scale bar = 10 mm. **(B–D)** Show grain length, grain width, and TGW of mature seeds of NIP and two *OsNAC129*-OE lines, respectively. Data are means ± SD of three replicates. **(E,F)** Show the AAC and TSC of NIP and the two *OsNAC129*-OE lines, respectively. Data are means ± SD of five replicates. ^**^p < 0.01, ^***^p < 0.001 as determined by Student’s *t*-test.

To further verify the functions of *OsNAC129* in rice seed development and plant growth, we also generated transgenic plants overexpressing the full length *OsNAC129* gene (including ~2 kb promoter sequence upstream of the ATG, the ORF, and ~1 kb downstream of the stop codon) in the NIP background (hereafter referred as OE plants; Supplementary Figure 1E). The results of a phenotypic analysis of OE plants showed that, compared with WT, grain width rather than grain length was reduced in the OE plants, leading to a decrease in grain weight (Figures 3A–D). These results further confirmed that *OsNAC129* plays a negative role in the regulation of grain size. Moreover, the AAC was reduced but TSC was unchanged in the seeds of OE plants compared with WT (Figures 3E,F). Above all, these results suggest that *OsNAC129* simultaneously and negatively regulates grain size and starch biosynthesis, especially AAC.

Additionally, we also checked the vegetative growth of *OsNAC129*-OE plants. In contrast to *osnac129* mutant plants, shoots and roots of OE seedlings were shorter than in the WT (Supplementary Figures 3A–C). Moreover, plant height was also reduced at maturity in the OE plants (Supplementary Figures 3D–F). These results show conclusively that *OsNAC129* plays negative roles in the regulation of grain size, starch synthesis, and plant height.

### Expression of Grain Size and Starch Biosynthesis-Related Genes Is Altered in Both *osnac129* Mutant and *OsNAC129*-OE Plants

**Figure 4:**
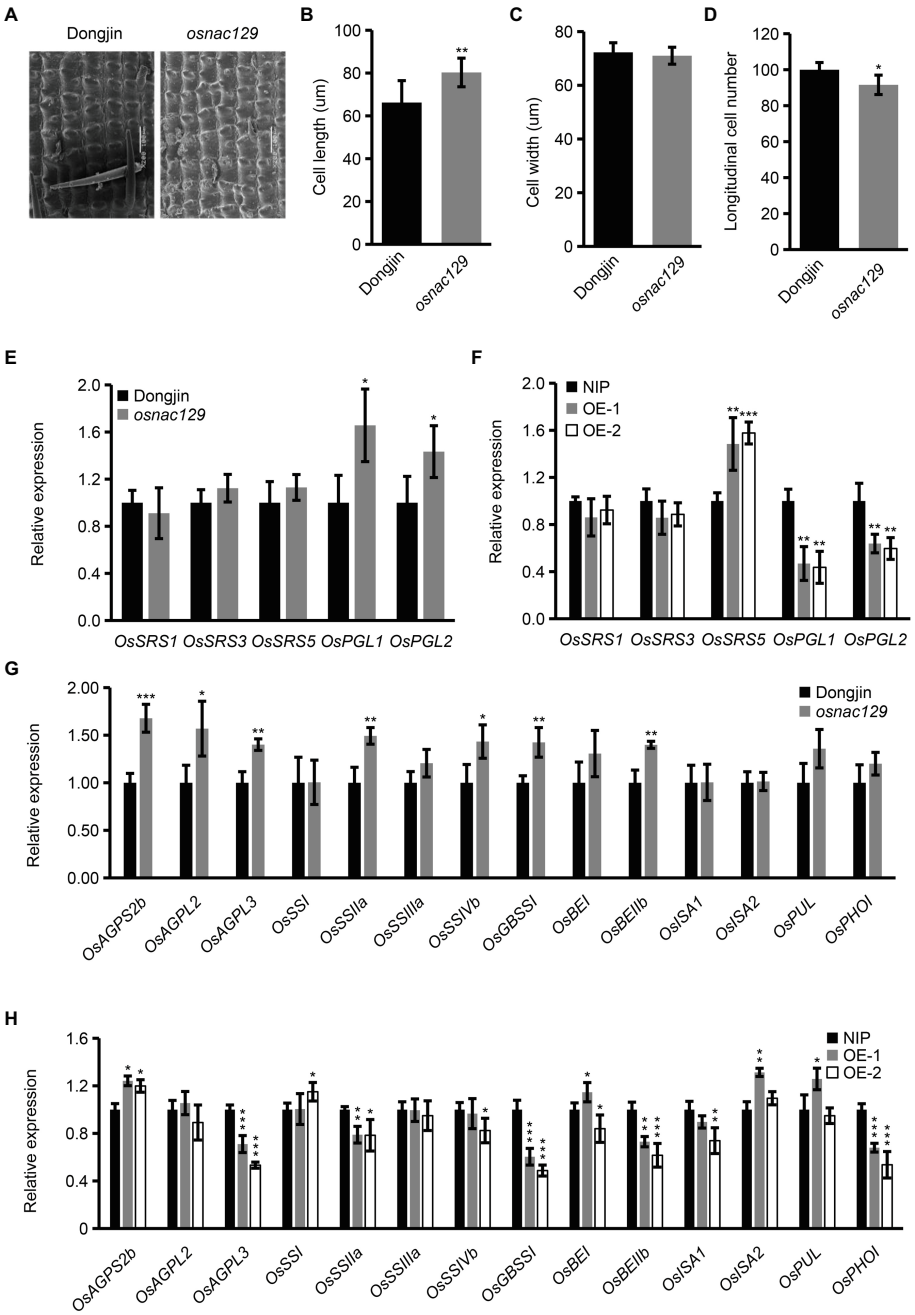
*OsNAC129* simultaneously regulates cell elongation and starch biosynthesis in rice seeds. **(A)** Observation of the outer epidermal cells of WT and *osnac129* mutant lemmas by SEM. Scale bars = 100 μm. **(B–D)** Show the cell length, cell width, and cell number measurement of the outer epidermal cells of the lemma. Data are means ± SD of three replicates (each replicate consisted of at least 30 cells) in **(B,C)**, and four replicates in **(D)**; ^**^p < 0.01, ^***^p < 0.001 as determined by Student’s *t*-test. **(E,F)** Show the relative expression determined by qRT-PCR of genes reported to be related to grain size by controlling cell elongation in seeds from the WT, *osnac129* mutant, and OE plants at 7 DAF. *UBQ10* was used as the internal control for normalization of gene expression. Data are means ± SD of four replicates. **(G,H)** Show qRT-PCR determination of expression of starch synthase-encoding genes in seeds of the WT, *osnac129*, and OE plants at 7 DAF. *UBQ10* was used as the internal control. Data are means ± SD of four replicates; ^*^p < 0.05, ^**^p < 0.01, and ^***^p < 0.001 as determined by Student’s *t*-test.

The above results showed that *OsNAC129* negatively regulates grain size (Figures 2A–E, 3A–D). In order to discover the regulatory mechanism, we observed cell differences in mature seeds of the *osnac129* mutant and WT using SEM. The results showed that cell length rather than cell width was increased in the *osnac129* mutant compared with WT, while the longitudinal cell number was reduced (Figures 4A–D), implying that *OsNAC129* regulates grain length by controlling cell elongation and division. At present, several genes, such as *OsSRS1*, *OsSRS3*, *OsSRS5*, *OsPGL1*, and *OsPGL2*, have been shown to participate in the regulation of grain size by promoting cell expansion (Abe et al., 2010; Heang and Sassa, 2012a,b; Segami et al., 2012; Deng et al., 2015). Therefore, we asked whether *OsNAC129*-regulated grain size depends on one or more of these genes. We found that, of these genes, the expression levels of *OsPGL1* and *OsPGL2* were significantly upregulated in *osnac129* seeds (Figure 4E), suggesting that the cell expansion promoted by *OsNAC129* might partly depend on them. Consistent with this speculation, *OsPGL1* and *OsPGL2* were downregulated in the *OsNAC129*-OE plants (Figure 4F), implying that *OsNAC129* negatively regulates *OsPGL1* and *OsPGL2* expression.

In addition, the above results also showed that *OsNAC129* participates in starch biosynthesis. Thus, we hypothesized that the expression levels of genes encoding starch synthase are affected in the *osnac129* mutant and OE plants. qRT-PCR assays showed that several starch synthase genes such as *OsAGPS2b*, *OsAGPL2*, *OsAGPL3*, *OsSIIa*, *OsSSIVb*, *OsGBSSI*, and *OsSBEIIb* were upregulated in the *osnac129* mutant (Figure 4G). However, we found that several starch synthase genes including *OsAGPL3*, *OsSIIa*, *OsGBSSI*, *OsBEIIb*, and *OsPHOI* were downregulated in OE endosperm, although *OsAGPS2b* was upregulated (Figure 4H). These results further confirm that *OsNAC129* plays a negative role in regulation the expression of starch synthase genes. However, these results are contradictory in that they do not explain why the TSC decreased in *osnac129* mutant seeds (Figure 2H) but was not changed in seeds from the *OsNAC129*-OE plants (Figure 3F). These results suggest that there might be more complex regulatory mechanisms that act at the post-transcriptional and translational levels in starch synthase-encoding genes.

### *OsNAC129* Expression Is Exclusively Induced by ABA, and *OsNAC129* Participates in the BR Signaling Pathway

**Figure 5:**
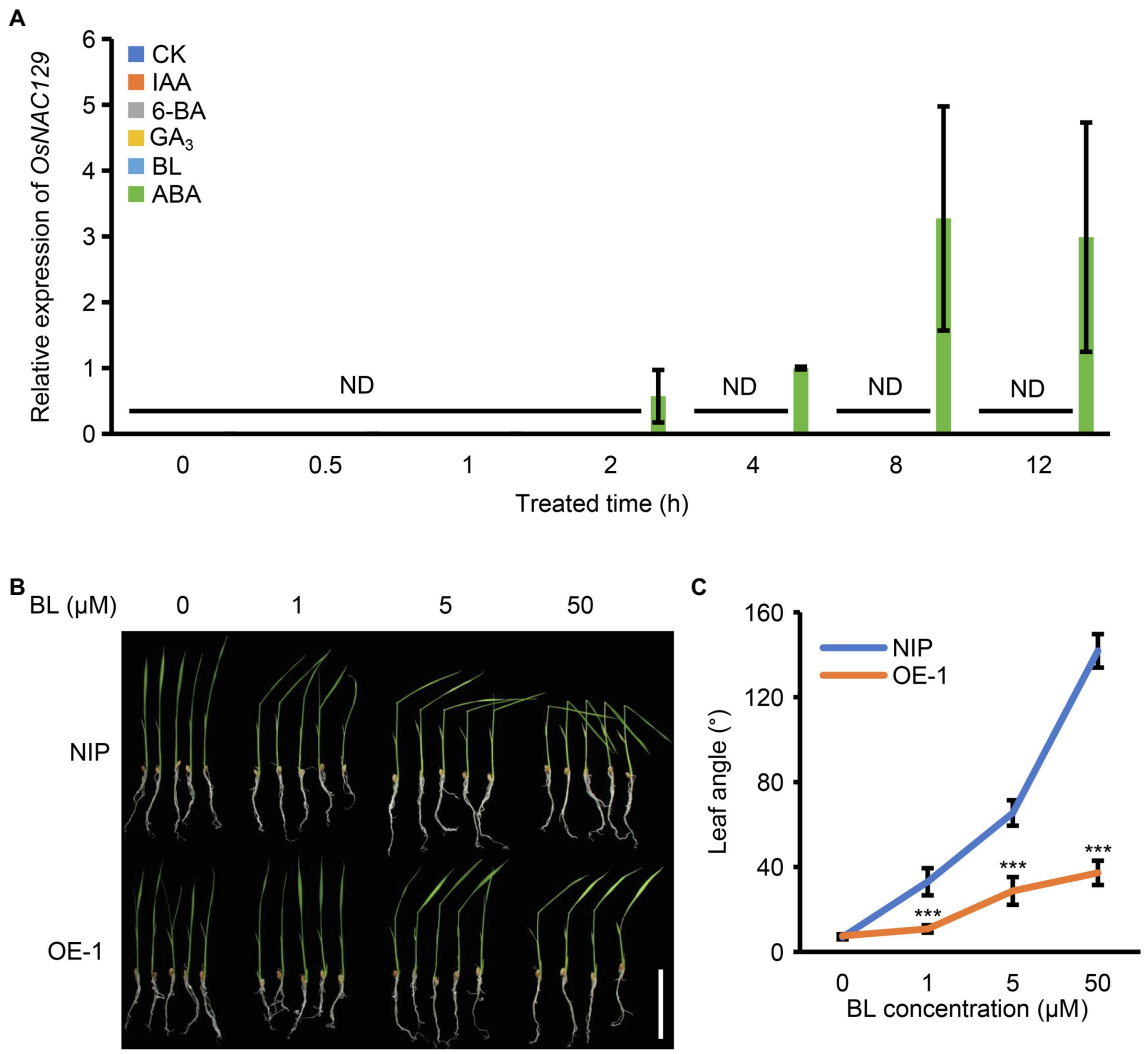
*OsNAC129* is an abscisic acid (ABA)-inducible and brassinolide (BR)-related gene. **(A)** *OsNAC129* was exclusively induced by ABA. About 1-week-old NIP seedlings were treated separately with IAA, 6-BA, GA_3_, BL, and ABA (50 μM of each phytohormone), and sterile water treatment was the control. Samples were collected, RNA was extracted, and the expression of *OsNAC129* was determined by qRT-PCR. Data are means ± SD of four replicates. ND, not detected. **(B)** About 2-week-old seedlings of WT and the *OsNAC129*-OE-1 line were treated with three concentrations of BL for 24 h, after which the leaf angles were imaged. Scale bar = 5 cm. **(C)** Leaf angles of WT and OE-1 seedlings measured by Image J after BL treatment. Data are means ± SD of 10 replicates. ^***^p < 0.001 as determined by Student’s *t*-test.

The above results suggest that the *osnac129* mutant has the potential to improve grain size in rice due to its increased grain length and grain weight; thus, we further explored the types of upstream signals that allow *OsNAC129* to participate in this process. Phytohormones are widely considered to be key regulatory signals that are involved in almost all aspects of plant growth and development. For example, indoleacetic acid (IAA) and BRs have been widely reported to positively regulate seed size in plants (Song, 2017; Figueiredo and Köhler, 2018; Cao et al., 2020). A very recent study showed that leaf-derived ABA regulates seed development by promoting the expression of starch synthesis genes (Qin et al., 2021). Therefore, we subsequently investigated the relationship between the expression of *OsNAC129* and phytohormones, such as IAA, 6-benzylaminopurine (6-BA), gibberellin A_3_ (GA_3_), brassinolide (BL), and ABA. About 1-week-old WT seedlings were treated with these phytohormones (50 μM of each) and the water treatment group was used as the control. qRT-PCR assays showed that the expression level of *OsNAC129* in seedlings was extremely low and almost undetectable, consistent with the expression profile determined previously (Figures 1A,B). However, after the hormone treatments, *OsNAC129* was exclusively and significantly induced by ABA but not by the others (Figure 5A), indicating that ABA could be the upstream signal that allows *OsNAC129* to participate in the corresponding regulation of a group of downstream genes.

Additionally, we showed that *OsNAC129* negatively regulates grain length, and that this regulation is partially dependent on two other grain size regulator genes, *OsPGL1* and *OsPGL2*. Both *OsPGL1* and *OsPGL2* were previously reported to be BR-related genes, even though *OsPGL1* was not BR-inducible (Heang and Sassa, 2012a,b; Jang et al., 2017; Jang and Li, 2017). We also found that the expression of *OsNAC129* could be detected in the lamina joints (Figures 1Bb,d), indicating a potential role in leaf bending/inclination regulation. Furthermore, promotion of leaf bending is representative of the BR response in cereal crops such as rice and maize. Thus, we wondered whether *OsNAC129* is a BR-related gene even though it is not BR-inducible, similar to *OsPGL1*. We treated 2-week old WT and *OsNAC129*-OE seedlings with a variety of concentration of exogenous BL and the angle of leaf inclination was measured. The results of this experiment showed that at the seedling stage, leaf angle was indistinguishable between WT and OE seedlings without BL treatment. In seedlings treated with increasing BL concentrations, leaf angle in WT seedlings increased significantly, but the leaf angle increased much more slowly in the OE plants (Figures 5B,C), indicating significantly reduced sensitivity to BL and a negative role for *OsNAC129* in the BR signaling pathway. Overall, these results suggest that transcription of *OsNAC129* is induced by ABA and that it participates in the BR signaling pathway.

## Discussion

In this study, we characterized a NAC TF-encoding gene, *OsNAC129*, that is, highly expressed in seeds, to examine its potential roles in regulating seed development and plant growth. Comprehensive phenotypic analysis and comparison of a T-DNA insertion mutant and *OsNAC129*-OE plants with WT revealed that *OsNAC129* simultaneously plays negative roles in regulating grain size, AAC, and plant height in rice. SEM observation of lemma outer epidermal cells indicated that *OsNAC129* inhibits grain length mainly by regulating cell elongation, and that this regulation is partially dependent on *OsPGL1* and *OsPGL2*. Furthermore, *OsNAC129* was shown to be a BR signaling pathway-related gene that acts as a negative regulator, although it was not BR-inducible, being similar to *OsPGL1* in that respect. Moreover, we also found that *OsNAC129* expression was exclusively induced by ABA in seedlings. Taken together, these results strongly suggest that *OsNAC129* regulates seed development and plant growth and participates in the BR signaling pathway.

*OsNAC129* was previously reported to be a seed-specific gene (Fang et al., 2008). To date, several of these NAC family TF genes such as *OsNAC20*, *OsNAC26*, *OsNAC127*, and *OsNAC129* had been confirmed to play important roles in the regulation of rice seed development (Wang et al., 2020; Ren et al., 2021). qRT-PCR assays confirmed that *OsNAC129* is highly expressed in immature seeds, peaking at 7 DAF, after which the mRNA levels gradually decline (Figure 1A). This pattern was finely matched with the expression profiles of genes encoding starch synthases and the grain filling process, similar to the expression of *OsNAC20*, *OsNAC26*, *OsNAC127*, and *OsNAC129* (Wang et al., 2020; Ren et al., 2021). However, histochemical GUS staining of transgenic plants expressing an *OsNAC129* pro::GUS construct further showed that there was still some GUS staining present in a few regions of some vegetative tissues, such as the leaves, leaf sheaths, lamina joints, and stem nodes (Figure 1B). These expression patterns are consistent with those reported by Ren et al. (2021), and imply that *OsNAC129* also functions in plant growth.

It is possible that the expression of *OsNAC129* in the lamina joints is related to BR signaling, because promotion of leaf inclination is one of the typical physiological effects of BRs (Wang et al., 2012b, 2014; Li et al., 2020). Several BR-related components that control leaf angle also show lamina joint-specific expression patterns, such as *OsBU1*, *OsPGL2*/*OsBUL1*, and *OsbHLH98* (Tanaka et al., 2009; Jang and Li, 2017; Guo et al., 2021). Furthermore, expression in the nodes and stems has been thought to relate to cell proliferation/expansion and nutrient transport. For example, our previous study reported that a spin-like gene, *OsRRM*, which modulates sugar transport, had a similar expression profile to *OsNAC129* in stems, and the *osrrm* mutant displayed reduced plant height and grain size, similar to the phenotypes observed in *OsNAC129*-OE plants (Liu et al., 2020). Very recently, another group reported that *OsNAC129* and its interactor *OsNAC127* do indeed participate in grain filling regulation by directly targeting sugar transporter genes including *OsMST6* and *OsSWEET4* (Ren et al., 2021). Thus, in our study, we could not rule out the possibility that *OsNAC129* might also participate in regulating sugar or other nutrient transport, and it would be worthwhile to further explore the mechanism. However, we did not observe the incomplete grain filling phenotype in *osnac129* mutants and *OsNAC129*-OE plants in this study, probably due to the use of different cultivars [Ren et al. (2021) used ZH11, while we used “Dongjin” and “Nipponbare”], planting conditions, and phenotypes.

Ren et al. (2021) showed that *OsNAC129* functions in grain filling. In our study, we showed that loss-of-function of *OsNAC129* led to multiple phenotypes, including increased grain length, grain weight, AAC, and plant height, but reduced TSC (Figures 2A–H; Supplementary Figures 2A–F). Moreover, overexpression of *OsNAC129* resulted in almost opposite phenotypes such as reduced grain width (instead of grain length), grain weight, AAC, and plant height, but slightly increased TSC (Figures 3A–F; Supplementary Figures 3A–F). These results provide evidence that *OsNAC129* plays a negative role in regulating grain size, AAC, and plant height. Further observation of the cells in *osnac129* and WT seeds revealed that the increase in grain length was caused by lemma cell elongation rather than cell proliferation (Figures 4A–D). qRT-PCR determination of the expression of several known genes associated with grain size through cell elongation control suggested that grain size regulation by *OsNAC129* probably and depends partially on *OsPGL1* and *OsPGL2* (Figures 4E,F). *OsPGL1* and *OsPGL2* were previously reported to encode two atypical bHLH proteins that positively regulate grain length by heterodimerizing with a typical bHLH protein, APG, and inhibiting its activity. Overexpression of *OsPGL1* and *OsPGL2*/*OsBUL1* and knock-down of *APG* expression gave plants that produced longer seeds than WT, similar to the grains produced by the *osnac129* mutant (Heang and Sassa, 2012a,b; Jang et al., 2017; Jang and Li, 2017). Moreover, *OsPGL1* was shown to be a BR-related gene that functions as a positive regulator, because plants overexpressing *OsPGL1* were hypersensitive to BL; however, its expression was not BL-inducible (Heang and Sassa, 2012a). The BR pathway is widely thought to be an important and classical pathway for the regulation of plant architecture (plant height, tiller number, and tiller angle), panicle morphology, and grain size (Müssig, 2005; Divi and Krishna, 2009; Fridman and Savaldi-Goldstein, 2013; Wei and Li, 2016; Zhang et al., 2019a; Ackerman-Lavert and Savaldi-Goldstein, 2020; Nolan et al., 2020). In this study, we found that *OsNAC129* is expressed in the lamina joints, stem nodes, and the spikelet, and controls plant height and grain shape simultaneously, which is highly correlated with plant phenotypes that are regulated by BRs. However, the OE plants showed significantly reduced sensitivity to exogenous BL (Figures 5B,C). Thus, *OsNAC129* seem to play a negative role in the BR pathway, antagonizing the functions of *OsPGL1* and *OsPGL2*.

In addition, qRT-PCR assays of genes that encode starch synthases in *osanc129* mutant and *OsNAC129*-OE plants revealed that *OsNAC129* repressed the expression of several of them, including *OsGBSSI* (*Wx*; Figures 4G,H). OsGBSSI, encoded by the *Wx* gene, is the only enzyme responsible for amylose synthesis in endosperm (Wang et al., 1990, 1995). Thus, the AAC was increased in grains from the *osnac129* mutant, but was reduced in OE plant grains compared to WT (Figures 2G, 3E). However, TSC was reduced in the *osnac129* mutant (Figure 2H). There are two hypotheses that may explain this phenomenon: (1) *OsNAC129* could also participate in regulating the synthesis of other storage molecules such as storage proteins; thus, loss-of-function of *OsNAC129* increased the content of not only starch but also of other substances. For instance, several TF genes including *OsNAC20*, *OsNAC26*, *ZmNAC128*, *ZmNAC130*, and *O2* have recently been reported to participate in regulating both starch and protein synthesis (Zhang et al., 2016, 2019b; Wang et al., 2020). (2) Starch synthases are regulated by complex mechanisms including the transcription level, post-transcription level, translation level, and protein interactions and modifications. For example, OsNAC20 and OsNAC26 can directly bind to the promoters and activate the expression of *AGPS2b*, *AGPL2*, and *OsSBEI*; however, the activities of AGPase and SBE were unchanged (Wang et al., 2020). Thus, it would be worthwhile to further explore the role of *OsNAC129* in the regulation of other storage substances in seeds and relative changes in the activity of starch synthases. It has been reported that OsNAC129 directly and negatively regulates the expression of the sugar transporter encoding genes *OsMST6* and *OsSWEET4* during grain filling (Ren et al., 2021). Further analysis of whether starch-synthesis enzyme-coding genes are the direct target of OsNAC129 will help to clarify the molecular mechanism by which *OsNAC129* regulates of starch synthesis.

In addition to the strong evidence showing that *OsNAC129* participates in the BR signaling pathway, we found that transcription of *OsNAC129* is exclusively induced by ABA in seedlings, indicating a potential role for this gene in the ABA signaling pathway. Recently, it has been reported that leaf-derived ABA not only plays important roles in the stress response, senescence, and seed dormancy, but is also vital in promoting starch synthesis and grain filling (Qin et al., 2021). *OsNAC129* is also a heat-stress responsive gene and both the *osnac129* mutants and OE plants exhibit more sensitivity to heat stress (Ren et al., 2021). Therefore, we speculate that *OsNAC129* probably coordinates BR and ABA signals to regulate diverse biological processes such as starch synthesis, grain filling, plant growth, and the heat stress response.

## Data Availability Statement

The datasets presented in this study can be found in online repositories. The names of the repository/repositories and accession number(s) can be found in the article/**Supplementary Material**.

## Author Contributions

J-PG and X-LC conceived the project and designed the study. S-KJ, M-QZ, Y-JL, L-NX, S-WJ, S-LW, TS, R-AW, Q-QY, and TT performed experiments. S-KJ, M-QZ, X-LC, and J-PG analyzed and interpreted the data. S-KJ and J-PG wrote the manuscript. All authors contributed to the article and approved the submitted version.

## Funding

This work was supported by grants from Jiangsu Province Government [JBGS(2021)001], Guangdong Province Key Research and Development Program (2018B020202012), National Natural Science Foundation of China (31771754), The Independent Scientific Research Project funds of the Jiangsu Key Laboratory of Crop Genomics and Molecular Breeding (PLR202101), Hainan Yazhou Bay Seed Lab (B21HJ0220-07), Natural Science Foundation of Shanghai (19ZR1466400), China Postdoctoral Science Foundation (2021M692723), and the Priority Academic Program Development of Jiangsu Higher Education Institutions.

## Conflict of Interest

The authors declare that the research was conducted in the absence of any commercial or financial relationships that could be construed as a potential conflict of interest.

## Publisher’s Note

All claims expressed in this article are solely those of the authors and do not necessarily represent those of their affiliated organizations, or those of the publisher, the editors and the reviewers. Any product that may be evaluated in this article, or claim that may be made by its manufacturer, is not guaranteed or endorsed by the publisher.

## Footnotes

^1^ http://www.ricedata.cn/gene/
