# Supplementary material for: OsNAC129 Regulates Seed Development and Plant Growth and Participates in the Brassinosteroid Signaling Pathway
Source: Front Plant Sci. 2022 May 16;13:905148. doi: 10.3389/fpls.2022.905148 (PMC9149566; doi:10.3389/fpls.2022.905148)
Supplement: Supplementary file 1 [file Data_Sheet_1.docx]

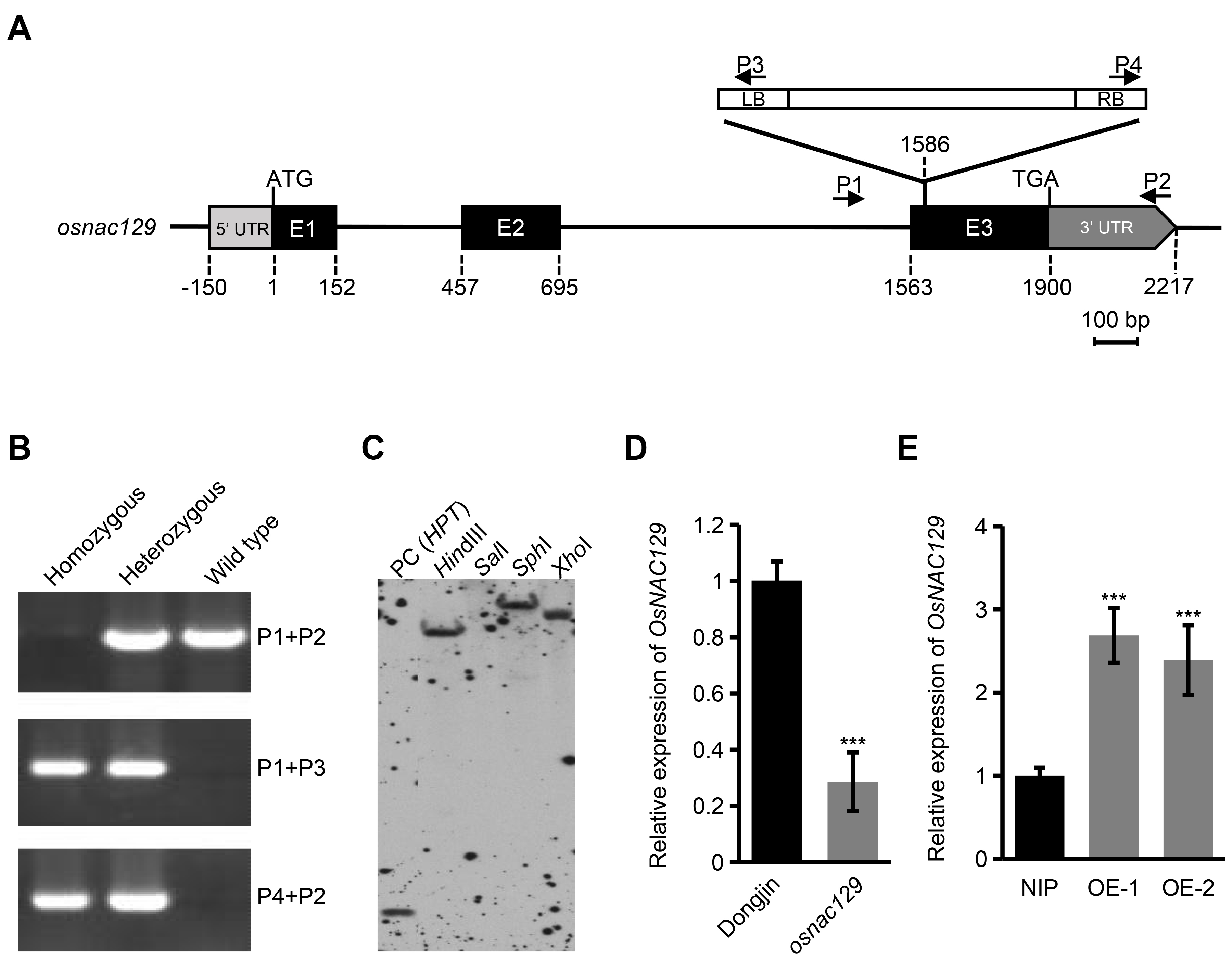


**Supplemental Figure 1**. Generation and identification of *OsNAC129*-related transgenic plants. **(A)** Schematic diagram of the *OsNAC129* gene showing the T-DNA insertion site in the *osnac129* mutant. Black boxes (E1, E2, and E3) represent the exons; Numbers indicate the positions of the nucleotides relative to the translational start site; P1, P2, P3, and P4 are the primers used in the identification of the T-DNA insertion site. LB, left border of T-DNA; RB, right border of T-DNA. Scale bar = 100 bp. **(B)** PCR screening of homozygous mutant plants. **(C)** Southern blot determination of the T-DNA insertion copy number. PC (*HPT*), is a DNA fragment of the hygromycin B phosphotransferase gene (*HPT*) obtained by PCR as a positive control. *Hin*dIII, *Sal*I, *Sph*I, and *Xho*l; genomic DNA extracted from *osnac129* mutant plants and digested individually with single restriction endonucleases. The hygromycin B phosphotransferase gene in the T-DNA was detected with a biotin labeled *HPT* probe. **(D-E)** qRT-PCR determination of *OsNAC129* gene expression in the *osnac129* mutant and the *OsNAC129*-overexpression plants (OE-1 and OE-2), respectively. *UBQ10* was used as the internal control**.** Data are means ± SD of four replicates. ****p* < 0.001 as determined by Student’s *t*-test.


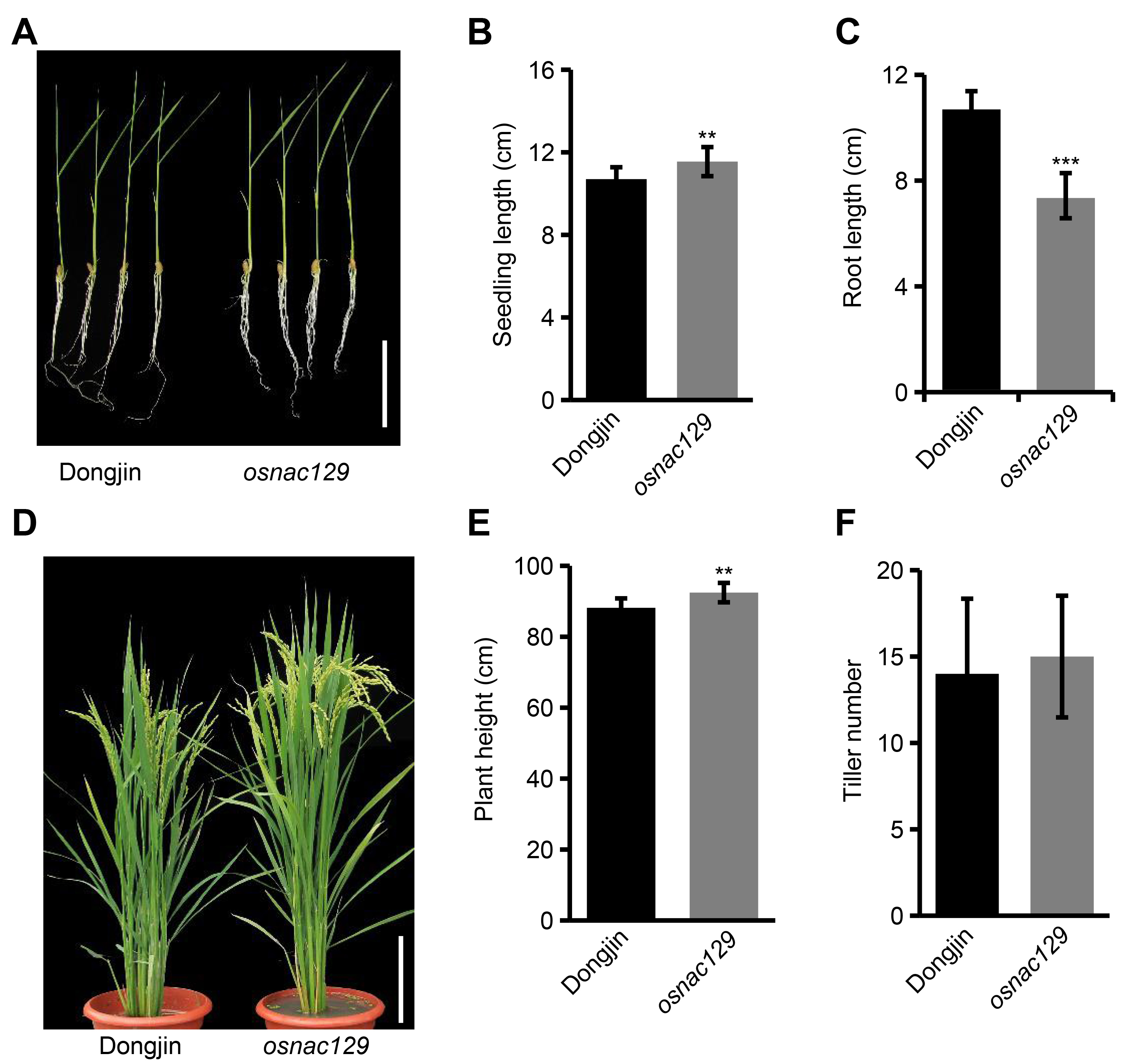


**Supplemental Figure 2**. Loss-of-function of *OsNAC129* increases plant height. **(A)** Two-week-old seedlings of WT and the *osnac129* mutant. Scale bar = 5 cm. **(B)** and **(C)** shoot lengths and root lengths in the WT and *osnac129* mutant seedlings two weeks after germination. Data are means ± SD of 10 replicates. **(D)** Plant architecture of WT and *osnac129* mutant plants at the grain filling stage. Scale bar = 20 cm. **(E)** and **(F)** plant height and tiller numbers in WT and *osnac129* mutant plants at the grain filling stage. Data are means ± SD of 12 replicates. ***p* < 0.01, ****p* < 0.001 as determined by Student’s *t*-test.


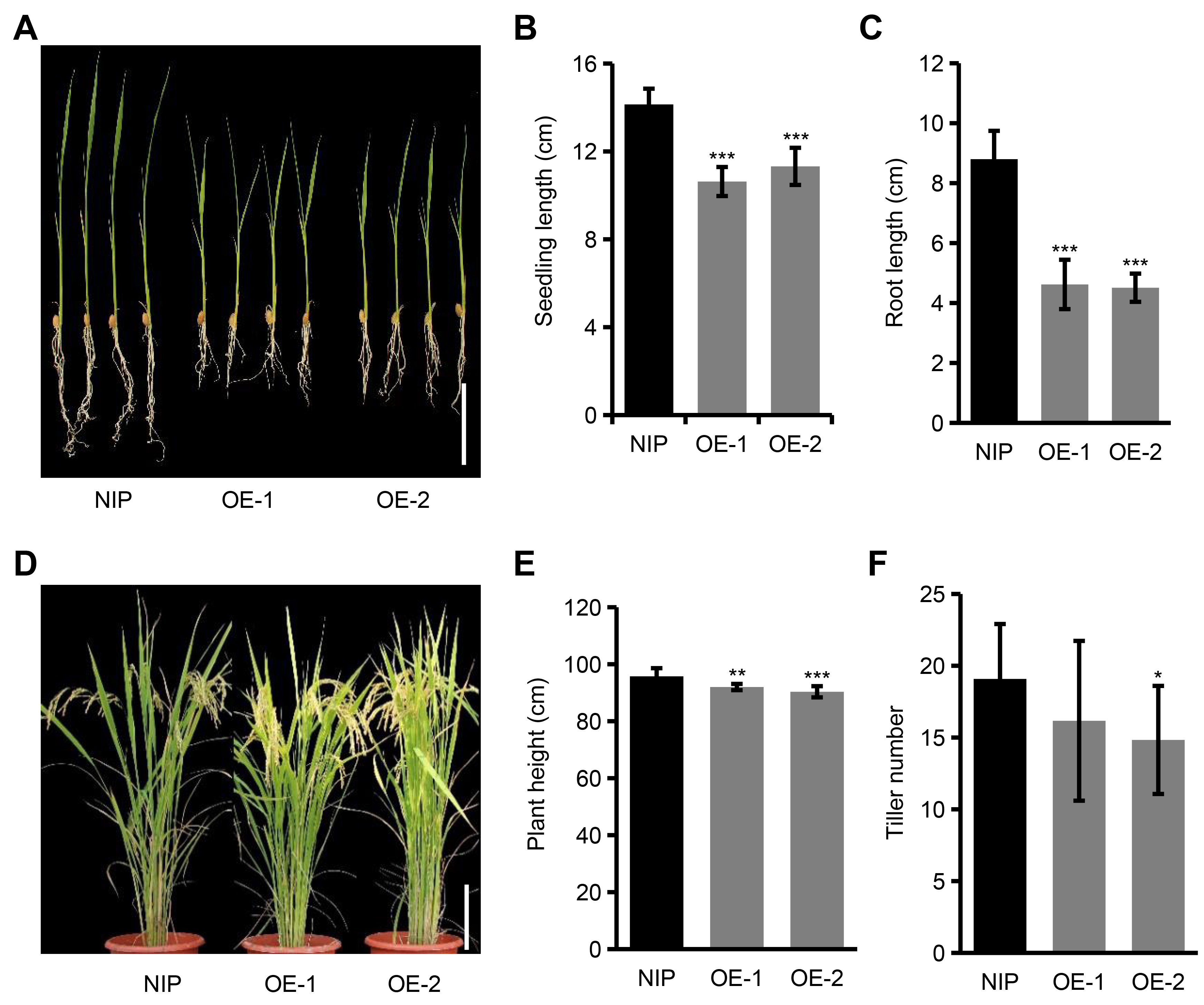


**Supplemental Figure 3**. Overexpression of *OsNAC129* inhibits plant growth. **(A)** Two-week-old seedlings of WT and two *OsNAC129*-OE transgenic lines. Scale bar = 5 cm. **(B)** and **(C)** shoot and root lengths of WT and *OsNAC129*-OE seedlings at two weeks after germination. Data are means ± SD of 10 replicates. **(D)** Plant architecture of WT and *OsNAC129*-OE plants at the grain filling stage. Scale bar = 20 cm. **(E)** and **(F)** plant height and tiller numbers in WT and *OsNAC129*-OE plants at the grain filling stage. Data are means ± SD of 12 replicates. **p* < 0.05, ***p* < 0.01, ****p* < 0.001 as determined by Student’s *t*-test.

**Supplemental Table 1**. Names and DNA sequences of the oligonucleotide primers used in this study.

| **Cloning and gene identification** | **Forward primer (5'→3')** | **Reverse primer (5'→3')** |
| --- | --- | --- |
| *OsNAC129* promoter | GGCCAGTGCCAAGCTTTGTGATTGATGCTGCTGATAACC | GACCACCCGGGGATCCATGGGAGTGGAAAGTACAACCC |
| *OsNAC129* genomic DNA | CGGTACCCGGGGATCCTGTGATTGATGCTGCTGATAACC | GGCCAGTGCCAAGCTTCTGCTCTGCCTGATTGTCGT |
| P1/P2 | TCATTTTCCGACATCAGGAAG | ACGCACACACACACACACAC |
| P4/P3 | TTGGGGTTTCTACAGGACGTAAC | CTAGAGTCGAGAATTCAGTACA |
| *HPT* | GCTTTCAGCTTCGATGTAGGAGG | TTTCCACTATCGGCGAGTACTTC |
| **qRT-PCR assay** | **Forward primer (5'→3')** | **Reverse primer (5'→3')** |
| *OsACTIN1* | CAGCCACACTGTCCCCATCTA | AGCAAGGTCGAGACGAAGGA |
| *OsUBA10* | TGGTCAGTAATCAGCCAGTTTGG | GCACCACAAATACTTGACGAACAG |
| *OsNAC129* | GCTCAAAACCACCCTGCAAC | CGACAACGGTTTCCACAAGG |
| *OsAGPS2b* | AACAATCGAAGCGCGAGAAA | GCCTGTAGTTGGCACCCAGA |
| *OsAGPL2* | AGTTCGATTCAAGACGGATAGC | CGACTTCCACAGGCAGCTTATT |
| *OsAGPL3* | AAGCCAGCCATGACCATTTG | CACACGGTAGATTCACGAGACAA |
| *OsGBSSI* | AACGTGGCTGCTCCTTGAA | TTGGCAATAAGCCACACACA |
| *OsSSI* | GGGCCTTCATGGATCAACC | CCGCTTCAAGCATCCTCATC |
| *OsSSIIa* | GCTTCCGGTTTGTGTGTTCA | CTTAATACTCCCTCAACTCCACCAT |
| *OsSSIIIa* | GCCTGCCCTGGACTACATTG | GCAAACATATGTACACGGTTCTGG |
| *OsSSIVb* | ATGCAGGAAGCCGAGATGTT | ACGACAATGGGTGCCAAGAT |
| *OsSBEI* | TGGCCATGGAAGAGTTGGC | CAGAAGCAACTGCTCCACC |
| *OsSBEIIb* | ATGCTAGAGTTTGACCGC | AGTGTGATGGATCCTGCC |
| *OsISA1* | TGCTCAGCTACTCCTCCATCATC | AGGACCGCACAACTTCAACATA |
| *OsISA2* | TAGAGGTCCTCTTGGAGG | AATCAGCTTCTGAGTCACCG |
| *OsPUL1* | ACCTTTCTTCCATGCTGG | CAAAGGTCTGAAAGATGGG |
| *OsPHOI* | TTGGCAGGAAGGTTTCGCT | CGAAGCCTGAAGTGAACTTGCT |
| *OsSRS1* | CCAGTTGAGCGTTTCCTCTG | GGCTCATGTTGGCAAGATTG |
| *OsSRS3* | CTCTTCTATGGAACCTGACAG | CTGAGAAGCTGAAGCAGATG |
| *OsSRS5* | ATGAGGGAGTGCATCTCGAT | CAAGATCGACGAAGACAGCA |
| *OsPGL1* | ATGTCAAGCCGGAGGTCAC | GATCGCTGATTTGCTCGTCG |
| *OsPGL2* | ATGTCGAGCAGAAGGTCGTC | TCAGGAGCGGAGGATGCTGC |
